# In silico-driven analysis of the *Glossina morsitans morsitans* antennae transcriptome in response to repellent or attractant compounds

**DOI:** 10.7717/peerj.11691

**Published:** 2021-07-01

**Authors:** Consolata Gakii, Billiah Kemunto Bwana, Grace Gathoni Mugambi, Esther Mukoya, Paul O. Mireji, Richard Rimiru

**Affiliations:** 1 Department of Mathematics, Computing and Information Technology, University of Embu, Embu, Eastern, Kenya; 2 School of Computing and Information Technology, Jomo Kenyatta University of Agriculture and Technology, Nairobi, Nairobi, Kenya; 3 Department of Biological Sciences, University of Embu, Embu, Eastern, Kenya; 4 Biotechnology Research Center, Kenya Agricultural & Livestock Research Organization, Nairobi, Nairobi, Kenya

**Keywords:** Association rule mining, Co-expression network, RNASeq data, Discretization, In silico analysis

## Abstract

**Background:**

High-throughput sequencing generates large volumes of biological data that must be interpreted to make meaningful inference on the biological function. Problems arise due to the large number of characteristics *p* (dimensions) that describe each record [*n*] in the database. Feature selection using a subset of variables extracted from the large datasets is one of the approaches towards solving this problem.

**Methodology:**

In this study we analyzed the transcriptome of *Glossina morsitans morsitans* (Tsetsefly) antennae after exposure to either a repellant (δ-nonalactone) or an attractant (ε-nonalactone). We identified 308 genes that were upregulated or downregulated due to exposure to a repellant (δ-nonalactone) or an attractant (ε-nonalactone) respectively. Weighted gene coexpression network analysis was used to cluster the genes into 12 modules and filter unconnected genes. Discretized and association rule mining was used to find association between genes thereby predicting the putative function of unannotated genes.

**Results and discussion:**

Among the significantly expressed chemosensory genes (FDR < 0.05) in response to Ɛ-nonalactone were gustatory receptors (GrIA and Gr28b), ionotrophic receptors (Ir41a and Ir75a), odorant binding proteins (Obp99b, Obp99d, Obp59a and Obp28a) and the odorant receptor (Or67d). Several non-chemosensory genes with no assigned function in the NCBI database were co-expressed with the chemosensory genes. Exposure to a repellent (δ-nonalactone) did not show any significant change between the treatment and control samples. We generated a coexpression network with 276 edges and 130 nodes. Genes CAH3, Ahcy, Ir64a, Or67c, Ir8a and Or67a had node degree values above 11 and therefore could be regarded as the top hub genes in the network. Association rule mining showed a relation between various genes based on their appearance in the same itemsets as consequent and antecedent.

## Introduction

Modern sequencing technologies generate large volumes of data from living cells under different physiological conditions and this ushered applied biology into the area of big data (Marx, 2013). As the dimensionality increases, the volume of data required for meaningful analysis also grows exponentially. This phenomenon was defined as a “curse of dimensionality” by Bellman (1957). Problems arise due to the large number of characteristics p (dimensions) that describe each record [$n$] in the database, that is, large [$n$] and small [*p*]. Various approaches have been used to reduce the dimensionality of bigdata. Feature selection is one of the approaches used to extract a subset of features (or variables) from the large datasets while maintaining as much information as possible (Yousef, Allmer & Khalifa, 2016). Another dimensionality reduction technique is data discretization and association rule mining. Discretization is used to convert continuous variables such as read counts from RNASeq data to a discrete format (Gallo et al., 2016). This reduces data noise and computational resources (Beleut et al., 2016).

Differential gene expression analysis is also a dimensionality reduction strategy. It deals with analysis and interpretation of variation in transcription levels of various genes in a cell across different samples or conditions (Nia et al., 2020). The method relies on different expression metrics e.g., non-parametric generalized linear models, independent sample *t*-tests, and log2 fold changes. However, identification of genes or pathways involved is problematic because genes act in concert rather than alone (Nacu et al., 2007). Biological interactions among genes are referred to as gene networks. A gene co-expression network has been defined as an undirected graph where nodes (genes) and edges connect significantly correlated features (Stuart et al., 2003; Gardner et al., 2003). Interactions between genes form a functional module and eventually different gene modules show varying levels of interaction (Gonzalez-Dominguez & Martin, 2017; Zhang et al., 2019). Since biological networks are usually complex, algorithms that utilize network theory have been extremely useful in deciphering valuable molecular interactions at the cell level (Fiscon et al., 2018).

Weighted gene co-expression network analysis (WGCNA) is a popular algorithm that constructs a network based on the pairwise correlations between gene expression levels (Zhang & Horvath, 2005; Langfelder & Horvath, 2008). The algorithm uses power law distribution to generate a scale-free network (Abbassi-Daloii, Kan & Raz, 2020). Genes that are highly interconnected and probably share a similar biological function are defined as a gene module (Roy, Bhattacharyya & Kalita, 2014). Co-expressed genes are grouped into increasingly large modules using hierarchical clustering algorithms (Abbassi-Daloii, Kan & Raz, 2020). Network metrics such as node degree (Deng, Zhu & Huang, 2015), betweenness (Wang, Hernandez & Van Mieghem, 2008) and cluster coefficient (Watts & Strogatz, 1998) are then used to explain which proteins are most important and why (Vella et al., 2017).

Gene co-expression network analyses have been widely used to complement other methods used in gene expression studies. For example, WGCNA was used by Morandin et al. (2016) to explain that there exist conserved pathways amongst 16 species of ants that exhibit reproductive division of labor. Using network analysis, Orsini et al. (2018) highlighted the role of metabolic and cell signaling genes in relation to stress response in the crustacean *Daphnia magna*. Kaur et al. (2019) compared the behavior and brain transcriptomes of *Temnothorax longispinosus* and identified enrichment in the WGCNA module of genes positively correlated with parasite prevalence and negatively with host attacks. Smith et al. (2018) used WGCNA to identify five co-expression modules that indicated a correlation between pupal stage of *D. melanogaster and* Osiris genes that are essential for development and phenotypic plasticity. Clusters (modules) of highly correlated genes between larval and adult stages have also been identified by Finch et al. (2020) in an Antarctic midge using Weighted correlation network analysis. Manfredini et al. (2017) identified gene coexpression subnetworks that were responsible for each queen phenotype from the brain transcriptomes of *B. terrestris*. Smith & Moran (2020) used gene expression and WGCNA to show that there exists a metabolic tug-of-war between the aphid *Acyrthosiphon pisum* and *Buchnera aphidicola* which is a bacterial symbiont. In a study by Chen et al. (2019), DEGs that may regulate wing dimorphism in the house cricket *Acheta domesticus* were detected using WGCNA. More recently, Mwangi, Macharia & Bargul (2021) identified significantly enriched modules for genes that play key roles during the development of *Trypanosoma brucei* in tsetse flies.

Tsetse flies belong to the genus *Glossina* which has twenty-three species and eight sub-species (Leak, 1999; Krafsur, 2009). They use olfaction to seek for hosts, locate oviposition sites, search for mates, as well as detecting and escaping from potential predators. They have been shown to exhibit unique behavioral responses towards volatile short-range allomones (Gikonyo et al., 2002). Responses towards various synthetic blends of these compounds elicits an avoidance behavior in the flies meaning that the compounds behave as repellents (Gikonyo et al., 2003). δ-octalactone is the most effective repellent in the blends (Bett, Saini & Hassanali, 2015). Exposure of male *G. m. morsitans* to ε-nonalactone (attractant) or δ-nonalactone (repellent) elicits antennal molecular responses that includes canonical and non-canonical chemosensory as well as novel odor specific transcripts (Kabaka et al., 2020).

The aim of this study was to use an in silico driven analysis to elucidate the putative function of some of the non-chemosensory genes that are co-expressed with the chemosensory genes. Differential expression analysis and coexpression network analysis were applied on the RNAseq data to identify the genes that were upregulated or downregulated due to exposure to either a repellant (δ-nonalactone) or an attractant (ε-nonalactone) in compassion to the untreated controls. Association rule mining (ARM) was thereafter used to identify itemset patterns/associations in the differentially expressed gene sets. ARM is a useful market basket analysis algorithm described by Agrawal & Srikant (1994) whereby datasets are presented in a transaction format whereby a transaction ***t*** ***T*** ∈ ***D*** contains itemset ${X} \in {I}$ if $X \subseteq I$. Using this approach, we were able to predict the potential function of uncharacterized genes based on their association with those with an assigned biological function.

## Materials & methods

### Sample collection, RNA extraction and sequencing

The male *G. m. morsitans* flies used in this study were from a colony reared at Yale University insectary as described by Bateta et al. (2017). Feeding, exposure to the chemicals and RNA extraction was done as described (Kabaka et al., 2020). A pair of antennae were carefully hand-dissected from fifty flies in each treatment (attractant, repellent, or control) and replicate (1, 2 or 3) as described (Menuz et al., 2014). PCR amplicons were generated using tsetse fly specific *beta*-*tubulin* gene primers to confirm removal of the gDNA from total RNA (Bateta et al., 2017). Sequencing was done on Illumina HiSeq 2500 at Yale University Center of Genome Analysis (YCGA), New Haven, CT, USA. The raw transcriptome sequences have been deposited at the Sequence Read Archive (SRA) under study accession number PRJNA343267.

### Data preprocessing and differential gene expression analysis

Quality of the FastQ files was checked using FastQC v0.11.5 (Babraham Bioinformatics, 2013) software and the output cleaned using Trimmomatic software v0.38 (Bolger, Lohse & Usadel, 2014). Any contaminating rRNA reads in the data were removed using SortMeRna v2.0 (Kopylova, Noé & Touzet, 2012). The clean paired reads from different treatments and replicates were then separately mapped onto *G. m. morsitans* transcripts gene-set version 1.9 from Vectorbase or genome version 1.0 (Giraldo-Calderón et al., 2015) using STAR software v2.7.3a (Dobin et al., 2013). VectorBase provides reliable datasets that are community reviewed and regularly updated. Chemosensory proteins in these datasets have been annotated (Obiero et al., 2014; Macharia et al., 2016; Liu et al., 2012). Transcript mapping provided information on transcript specific mapping abundance (Mortazavi et al., 2008). Mapping reads to the genome was an additional quality control procedure that would get rid non-*G. m. morsitans* contaminants. The BAM files contained information on sequences aligned onto the transcripts, sorted by respective coordinates to facilitate downstream analyses. The number of reads (counts) aligned onto each transcript in the respective BAM files were quantified using Salmon software v1.2.1 (Patro et al., 2017). This analysis provided data on relative abundance of read from different treatments and replicates that mapped onto *G. m. morsitans transcripts*.

Differential expression analysis was done on the gene count matrix from Salmon using the DESeq2 R-package version 1.28.0 (Love, Huber & Anders, 2014). Default parameters for count data normalization as recommended (Conesa et al., 2016) were used to allow for control of log_2_ fold change shrinkage, custom p-value and fold change cut-offs. Genes were considered differentially expressed and retained for further analysis if the test statistics *p-value* (adjusted for false detection rate) (FDR) was less than 0.05 according to the method from Benjamini & Hochberg (1995). Since the antennae is functionally specialized for olfaction, and potentially enriched with associated canonical chemosensory gene transcripts (Kabaka et al., 2020), we separately probed for expression profiles of these transcripts, and isolated those with at least two-fold change in difference between the attractant or repellent and control treatments. We generated heatmaps for visualizing the relationships between the different treatments using the package Pheatmap *v*1.0.12 (Kolde, 2012) in R software (R Core Team, 2017).

### Co-expression analysis using WGCNA

WGCNA package is designed for clustering genes based on their expression profiles and therefore we used the gene lists generated from Salmon for weighted co-express analysis. Normalization was done by filtering out genes with counts less than 10 in more than 90% of samples since they are not informative and tend to introduce noise (Farhadian et al., 2021). Co-expression networks were generated using WGCNA using the Bioconductor R package v3.5.1 (Langfelder & Horvath, 2008). This algorithm calculates a similarity co-expression matrix using correlation for all genes defined as: ${s_{ij}} = \left| {{\rm cor}\left( {{x_{{i_\; }}},{x_j}} \right)} \right|$. An adjacency matrix was calculated by raising the co-expression similarity to a soft thresholding power beta $\beta$ defined as ${a_{ij}} = {p_{ower({S_{{\rm ij}}}}},\beta ) = {\left| {{s_{ij}}} \right|^\beta }$, where ${a_{ij}}$ represents the resulting adjacency which is a measure of the connection strengths. A $\beta$ of 12 was selected for construction of a gene co-expression network based on estimated scale-free topology as described (Langfelder & Horvath, 2008). A topological overlap matrix (TOM) was computed, converted into dissimilarity matrix and then hierarchical clustering used to generate a tree (dendrogram). Co-expression modules were detected using Dynamic Tree Cut (DTC) algorithm based on an edge height cut-off of 0.25, and a minimum module size of 30 genes. Relationship between different co-expression was explored using FlashClust function (Langfelder & Horvath, 2012) and a heatmap was used to visualize the correlation between the modules.

### Network analysis

Network visualization and centrality measure analysis were done using Cytoscape v3.7.2 (Shannon et al., 2003). The degree, betweenness centrality and clustering coefficient of the network were analyzed using network analyzer (a Cytoscape plugin) as described by Assenov et al. (2008). Degree $d\left( v \right)$ of a vertex v, in a network $G{\rm \; } = {\rm \; }\left( {V,{\rm \; }E} \right)$, counts the number of edges in E incident upon v. Given G, define f (d) to be the fraction of vertexes ${\rm \; }v{\rm \; } \in {\rm \; }V{\rm \; }$ with degree $d\left( v \right){\rm \; } = {\rm \; }d$. Genes having large degrees are referred to as hubs genes, indicating that they hold multiple genes/proteins together and have the highest potential to regulate the node $v$. Betweenness is a measure of the number of shortest paths that are connecting any two nodes (j, k). On the other hand, closeness is a measure of the ability of a node to interact with all other nodes, including the indirectly connected nodes. It is defined as:

$$\mathop \sum \limits_J^{{j_1} = \displaystyle{1 \over N}} h\left( {{ i},i} \right)$$

with $h\left( {{i},i} \right)$ being the shortest distance between gene ${\rm i}$ and ${\rm j}$. Finally, clustering coefficient is defined as the edge density of the neighborhood of node $i$ which is calculated as:

$${u_{i = \displaystyle{{{m_i}\left( {j,k} \right)} \over {{m_i}}}}}$$

where ${\rm \; }{m_i}\left( {j,k} \right)$ is the number of edges connecting nodes (j, k) neighboring node i *and m_i_* is the total number of visible edges of all the neighboring nodes of i that are fully connected.

### Discretization and association rule mining

Discretization is a data pre-processing step used in machine learning in order to transform continuous or numerical attributes into discrete ones (Kotsiantis & Kanellopoulos, 2006; Hacibeyoglu & Ibrahim, 2018). In this study, we used the equal frequency discretization method (Dougherty, Kohavi & Sahami, 1995) implemented in GEDPROTOOLS (Gallo et al., 2016) to transform the data from continuous to discrete values. Equal frequency discretization reduces the effect of outliers and collects similar values in the same interval (Hacibeyoglu & Ibrahim, 2018). Genes that were co-expressed in the network were retrieved for discretization using the steps outlined below:

**Input:** the continuous values of attribute and number if intervals ***A*** = {*a*_1_, *a*_2_,…, *a_n_*_−1_, *a_n_*}
  and number of intervals ***k***, where ***k*** > 0.
  **Step 1:** Sort all values of in ascending order,
  **Step 2:** Divide ***A*** by *k* intervals,
  **Step 3:** Create bins according to number of elements in each interval,
  **Step 4:** Determine boundaries of each interval by calculating the average value of the
  Maximum value of the current bin and the minimum value of the next bin,
  **Step 5:** The continuous values of ***A*** are transformed into discrete ones by determining
  the interval that they belong to,
  **Output: *A*** with discrete values

Two bins were created in step 3, with the final output being discretized measurements whereby a value of zero represented a gene that was under-expressed, while a value of one represented a gene that was overexpressed. In this context, the discretized data was used to identify frequent itemsets using the Apriori algorithm (Agrawal & Srikant, 1994) implemented in the R package arules ver. v1.6-4 (Hahsler et al., 2014). In association rule mining, a rule is typically described by three measures: support, confidence, and lift. These three represent the significance and interest of a rule. Support of a rule $X \Rightarrow Y$ is equal to the support of the itemset $X \cup Y$ and is defined as the probability of finding all the genes in sets *X*. Support of an itemset X is calculated as:

$$Suppor{t_D}\left( x \right) = \displaystyle{{\left| {\left\{ {T \in Dx \subseteq T} \right\}} \right|} \over {\left| D \right|}}$$

The confidence of rule $X \Rightarrow Y$ is the probability of finding all the differentially expressed genes in set *Y* as compared with the differentially expressed genes in set X. The confidence is calculated as:

$$Confidenc{e_D}\left( {x \Rightarrow y} \right) = \displaystyle{{Sup{p_D}\left( {X \cup Y} \right)} \over {sup{p_D}\left( X \right)}}$$

Lift measures the strength of the rule and varies in the interval [0, ∞]. Lift is defined as:

$$lift\left( {X \to Y} \right) = \displaystyle{{supp\left( {X \cup Y} \right)} \over {supp\left( X \right){\rm *}supp\left( Y \right)}}$$

We used a support value of 0.5 and a confidence value of 0.99. Support values greater than 0.5 gave zero rules while support values of less than 0.5 and confidence of less than 0.99 resulted in too many rules. We therefore filtered and retained only the rules that had a lift value ≥ 2.

### Gene set enrichment analysis

Gene Set Enrichment Analysis (GSEA) was done using WEB-based Gene SeT AnaLysis Toolkit (WebGestalt) as described by Wang et al. (2017). Using *Drosophila melanogaster* homologs as proxy to assess gene enrichment since the database of WebGestalt does not have *G. morsitans*, homologs. FDR corrected and p-value ranked *D. melanogaster* gene homologs of the differentially expressed *G. m. morsitans* genes were selected as input for the analysis using default parameters (5–2,000 Entrez Gene IDs, FDR < 0.05, 1,000 permutations and 20 categories with the outputted leading-edge genes) as described by Dębski et al. (2016). We separated significantly enriched non-redundant biological processes, cellular components, and molecular function Gene Ontology (GO) terms as identified by GSEA (Ashburner et al., 2000; Stark et al., 2006; The Gene Ontology Consortium, 2017).

## Results

### Differential expression in response to repellants (δ-nonalactone) or attractant (Ɛ-nonalactone)

**Figure 1:**
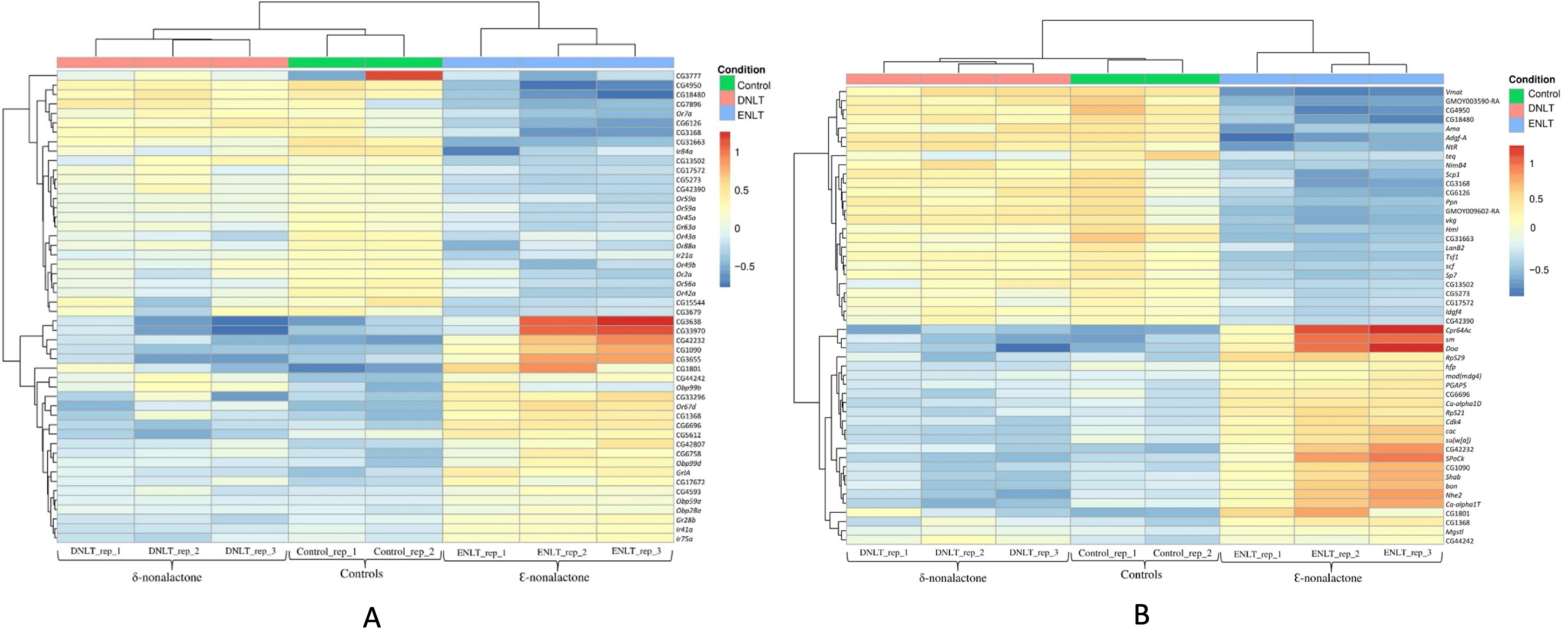
Top 50 chemosensory genes. Top 50 differentially expressed genes after exposure to repellant (δ-nonalactone) or attractant (Ɛ-nonalactone). (A) Top 50 chemosensory genes and associating genes with no assigned function. (B) Top 50 non-chemosensory genes and associating genes with no assigned function.

Raw reads per sample ranged between 23 and 73 million. Ribosomal RNA contamination levels were below 5% in all the samples indicating a near-perfect ribosomal RNA depletion before sequencing was done. The number of clean reads in the samples after preprocessing and quality filtering ranged between 11.1 M and 38.3 M. Reads that mapped to the genome were between 78% and 92%, while the remainder mapped to multiple locations, were not unique or had no features. During differential gene expression analysis, 2,097 low-count genes were filtered out leaving a total 10,921. Genes with a False discovery rate (FDR) < 0.05 were considered as being of biological significance (Von Der Weid et al., 2015). A set of 308 genes were identified as differentially expressed after exposure to either repellent (δ-nonalactone) or attractant (Ɛ-nonalactone) as compared to the control (no treatment). Figure 1A shows the top 50 significantly expressed chemosensory genes (FDR < 0.05) in response to Ɛ-nonalactone. Nine genes showed upregulation in flies exposed to an attractant (Ɛ-nonalactone). These are the Gustatory receptors (GrIA and Gr28b), ionotrophic receptors (Ir41a and Ir75a), odorant binding proteins (Obp99b, Obp99d, Obp59a and Obp28a) and the odorant receptor (Or67d). In addition, several non-chemosensory genes with no assigned function in the NCBI database were co-expressed with the chemosensory genes. In contrast, exposure to repellent (δ-nonalactone) did not show any significant change between the treatment and control samples. We identified 24 non-chemosensory that showed significant upregulation in response to Ɛ-nonalactone as shown in Fig. 1B. Six of these genes have no assigned function biological function and were also upregulated in the chemosensory geneset.

### Co-expression network analysis and hub genes identification

**Figure 2:**
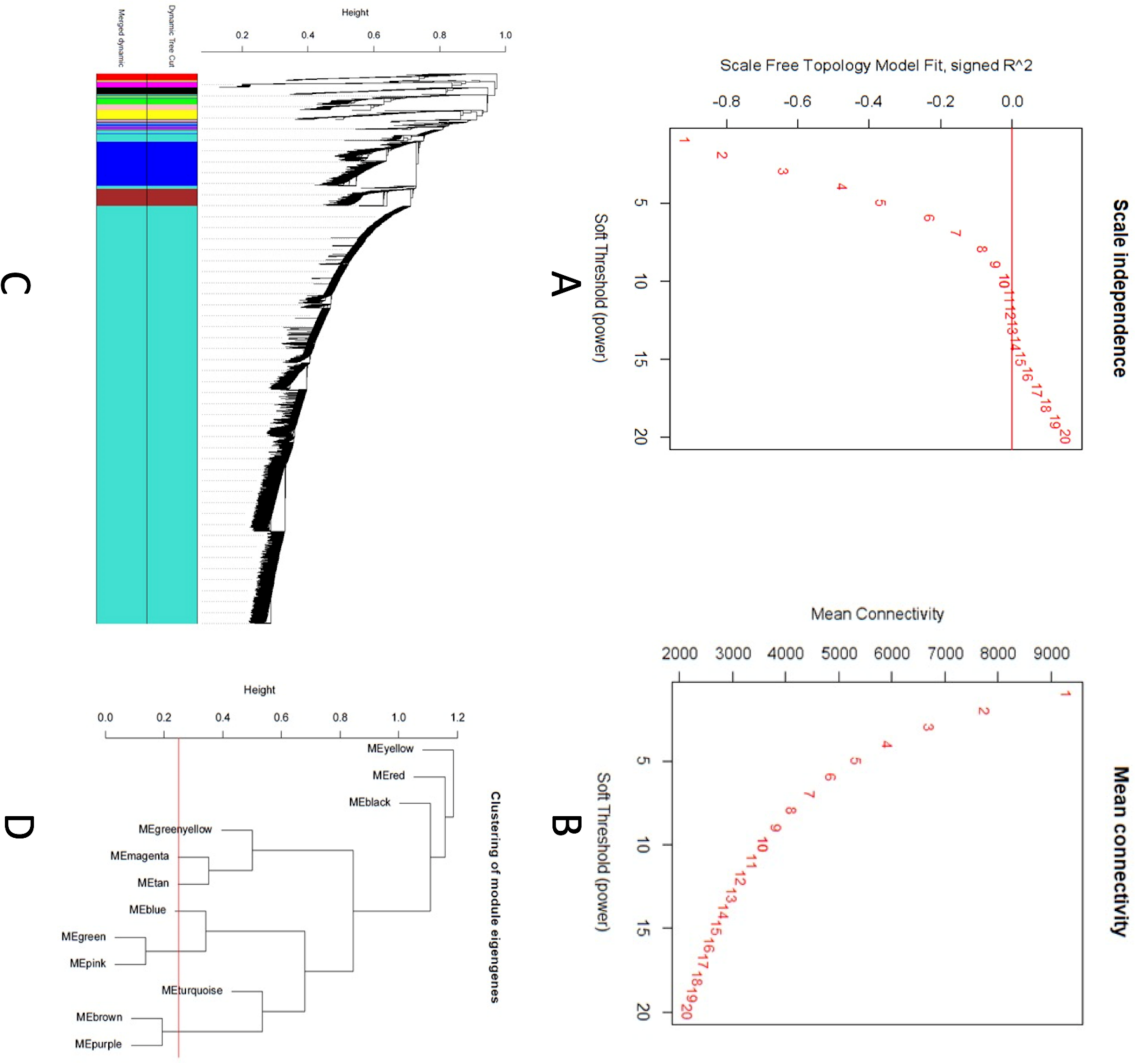
Co-expression network analysis using WGCNA. (A) Scale-free fit index versus soft-thresholding power. (B) Mean connectivity versus soft-thresholding power of 12. (C) Cluster dendrogram based on dissimilarity measures (1-TOM). The branches correspond to modules of highly interconnected groups of genes. Colors in the horizontal bar represent the modules. A total of 12 modules were identified. (D) Module network dendrogram constructed by clustering module eigengene distances. The red line shows the merging threshold.

A scale-free topology weighted gene network was constructed using WGCNA based on a soft thresholding power (β). From candidate powers of between 1–20, β = 12 returned a scale-free topology fit index of –0.1. An adjacency matrix based on the criterion of approximate scale-free topology is shown in Figs. 2A and 2B. Using the dynamic tree cutting algorithm, all the 11,808 genes were grouped into 12 modules, which ranged in size from 42 to 9,325 genes per modules (Figs. 2C and 2D).

**Figure 3:**
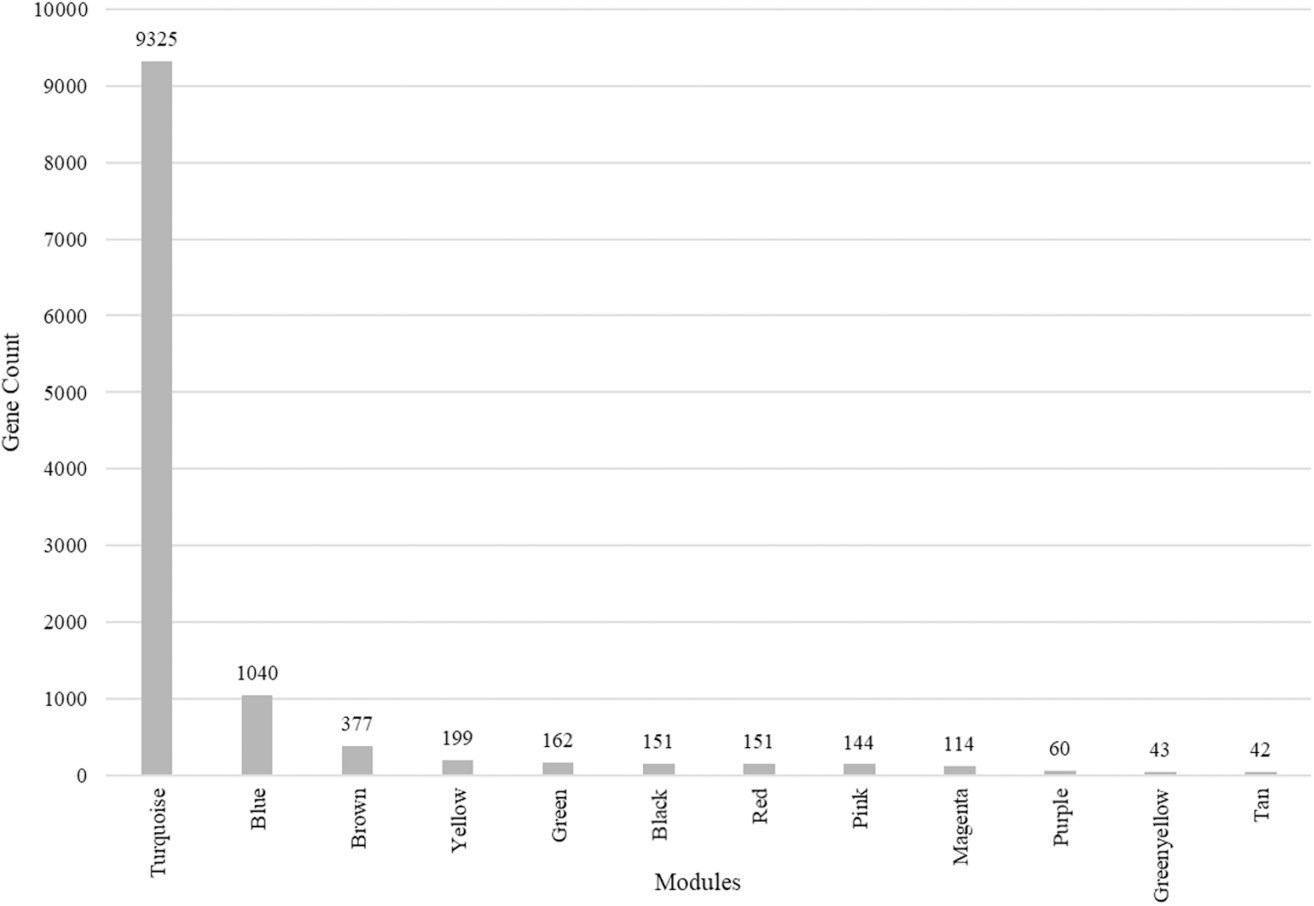
Modules identified along with the number of genes in each module. Total number of modules identified along with the number of genes in each module.

We identified 12 modules (Fig. 3) with the major modules being turquoise (*n* = 9,325 genes), blue (*n* = 1,040 genes), and brown (*n* = 377 genes).

### Correlation between modules

**Figure 4:**
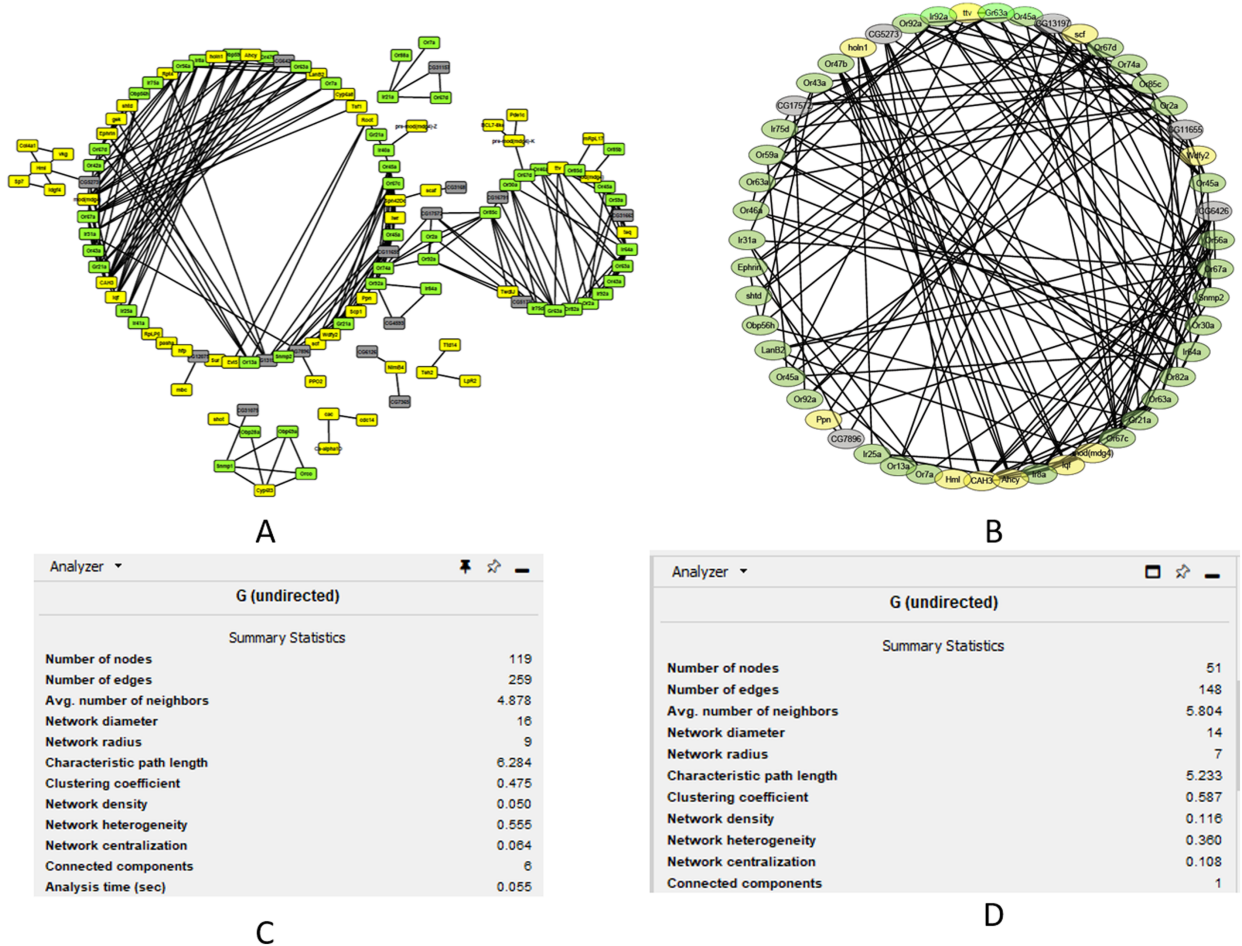
Co-expression networks for the genes in the turquoise, blue, brown and yellow modules. Co-expression networks analysis. (A) Subnetwork for genes in the turquoise, blue, brown and yellow modules. (B) Filtered co-expression network for the genes with a degree value greater than five. (C) Network summary statistics before filtering. (D) Network summary statistics after filtering.

We performed cluster analysis to identify whether chemosensory genes were evenly distributed with the 12 co-expressed modules. Using an edge height cut-off of 0.25, all modules were below the 75% similarity hence no modules were merged. Interestingly, most of the chemosensory genes (*n* = 83) were in the turquoise module which also had 77 non-chemosensory genes as well as 46 genes that have no assigned function yet. In the other modules, genes Or67d was identified in the blue module, Obp19b & Obp83g in the brown module, and Obp73a in the yellow module. Therefore, the turquoise module was interesting from the chemosensation point of view. We proceeded to probe if there exist relationships between genes in the turquoise, blue, brown and yellow modules by visualizing only the functionally annotated genes in a network (Figs. 4A and 4B). Chemosensory genes are depicted in green, non-chemosensory in yellow and those with unknown function in grey. We filtered out genes with degree value of less than 5 to reduce the size of the graph and got a final network with 51 nodes and 148 edges (Fig. 4B). Filtering changed the network density from 0.05 to 0.116 while overall clustering coefficient increased from 0.475 to 0.587 (Figs. 4C and 4D).

The degree, average shortest path length and clustering coefficient for the top genes with a node greater than 8 are shown in Table 1. Genes *CAH3*, *Ahcy*, *Ir64a*, *Or67c*, *Ir8a* and *Or67a* can be regarded as the top hub genes since they had degree values above 10. Fourteen of the top 20 genes are associated with chemosensation, which is an important biological function in insects. Clustering coefficient of hub gene nodes ranged between 0.29 and 0.68 and this is an indication that some parts of the network were more intricately connected than others.

**Table 1:** Network topology for the top genes.

No.	Gene symbol	Clustering coefficient	Degree	Betweenness centrality	
1	*Ahcy*	0.47	11	0.06	
2	*CAH3*	0.53	11	0.07	
3	*Ir64a*	0.38	10	0.1	
4	*Or67c*	0.44	10	0.2	
5	*Ir8a*	0.6	10	0.03	
6	*Or67a*	0.29	10	0.08	
7	*lqf*	0.56	9	0.02	
8	*mod(mdg4)*	0.58	9	0.01	
9	*Or30a*	0.31	9	0.24	
10	*Or45a*	0.5	9	0.11	
11	*Or85c*	0.36	9	0.32	
12	*CG11655*	0.57	8	0.18	
13	*Wdfy2*	0.61	8	0.03	
14	*Or82a*	0.39	8	0.05	
15	*Or2a*	0.54	8	0	
16	*Or67d*	0.32	8	0.22	
17	*Or45a*	0.46	8	0.2	
18	*Or63a*	0.68	8	0.02	
19	*Or56a*	0.46	8	0.09	
20	*Gr21a*	0.43	8	0.14	

### Association rule mining

Discretized and transformed gene expression data is presented in a transaction format where the samples represent transaction IDs and genes represent items. A minimum support of 0.5 and a confidence of 0.99 was used to generate 801 rules, which we further filtered using a lift of $\ge 2$ to empirically produce the best results. Lift values lower than 2 or support values less than 0.5 generated too many rules whereas when we used support values greater than 0.5, no rules were generated. Genes with no assigned biological function were of interest since we wanted to find out if association rule mining could help predict their function. We therefore arrowed down the rules that implied an association between known genes and those with no known function. Twenty-two representative rules are shown in Table 2.

**Table 2:** Association rules among genes that showed significant upregulation.

No.	Association rule	Set
1.	{Ir84a,Or2a,Or42a,Or56a} => {CG3679}	A
2.	{Or2a,Or42a,Or56a,Or49b} => {CG3679}	
3.	{Ir84a,Or42a,Or56a,Or49b} => {CG3679}	
4.	{Or88a,Gr63a,CG5273,CG17572} => {CG18480}	
5.	{CG4950,Or88a,Gr63a,CG5273} => {CG18480}	
6.	{Or88a,Gr63a,CG17572,CG31663} => {CG18480}	
7.	{Or88a, Gr63a, CG5273,CG17572} => {CG31663}	
8.	{CG4950, Or88a, Gr63a,CG5273} => {CG31663}	
9.	{CG4950,Or88a,CG18480,Gr63a} => {CG31663}	
10.	{CG4950,Or88a,Gr63a,CG31663} => {CG17572}	
11.	{Or88a,Gr63a,CG5273,CG31663} => {CG17572}	
12.	{Or88a,Gr63a,CG17572,CG31663} => {CG5273}	
13.	{CG4950,Or88a,Gr63a,CG17572} => {CG5273}	
14.	{CG4950,Or88a,CG18480,Gr63a} => {CG5273}	
15.	{Tsf1,Scp1,vkg,Adgf.A} => {CG6126}	B
16.	{Ppn,Sp7,NtR,Adgf.A} => {CG6126}	
17.	{vkg,Sp7,NtR,Adgf.A} => {CG6126}	
18.	{LanB2,Sp7,NtR,Adgf.A} => {CG6126}	
19.	{Sp7,NtR,Adgf.A,CG6126} => {CG3168}	
20.	{Ppn,NtR,Adgf.A,CG6126} => {CG3168}	
21.	{Idgf4,NtR,Adgf.A,CG6126} => {CG3168}	
22.	{Ppn,Sp7,Adgf.A,CG6126} => {CG3168}	

### Gene set enrichment analysis (GSEA)

**Figure 5:**
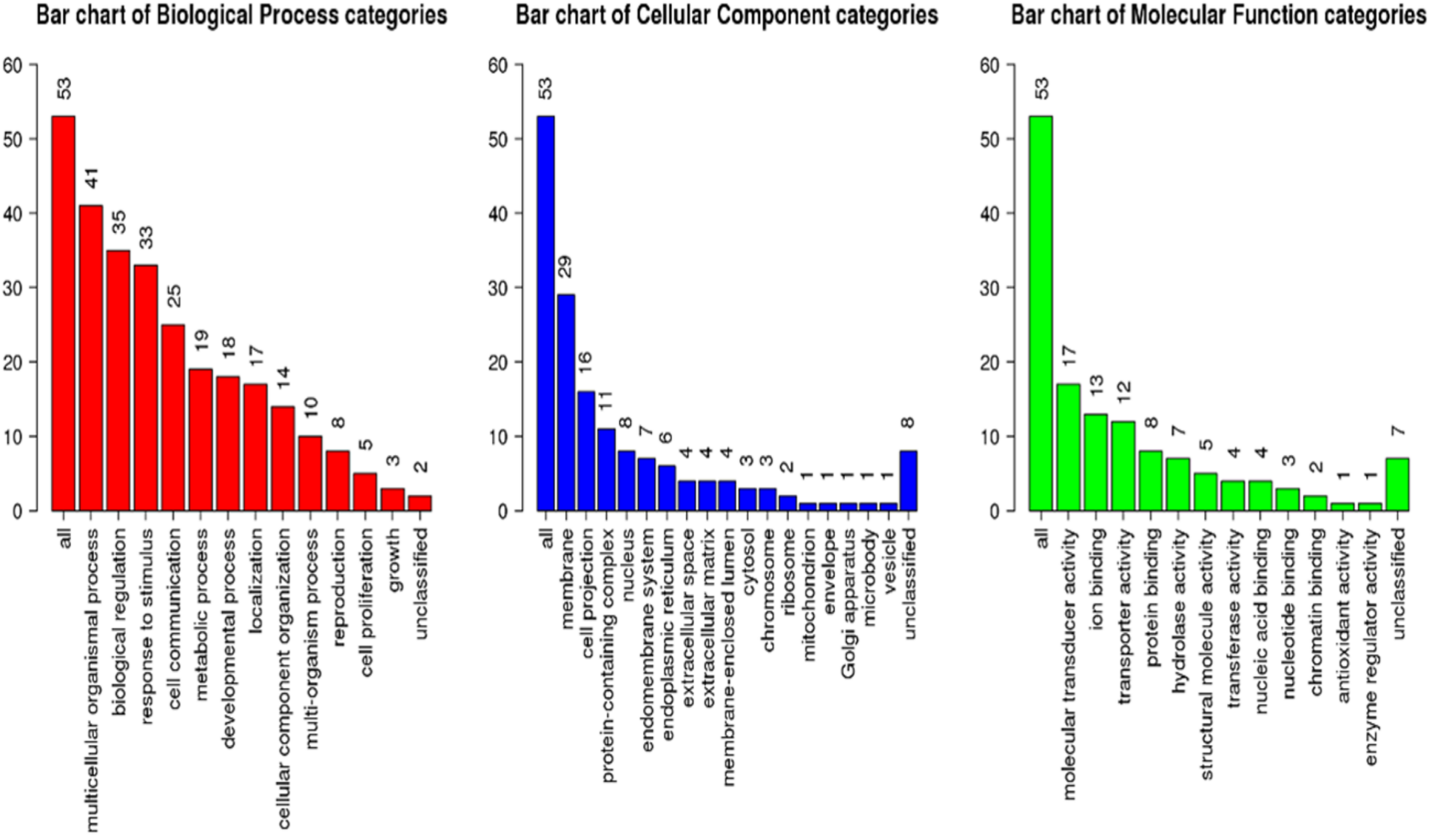
Geneset Enrichment Analysis (GSEA) of differentially expressed transcripts in *G. m. morsitans* antennae.

GSEA of the antennae transcripts revealed several enriched processes involved in detection of stimulus. GoSlim GO analysis component of the GSEA assigned most of the significantly expressed genes to various biological, cellular components and molecular function as shown in Fig. 5.

The top Enriched biological processes included biological regulation, response to stimulus, metabolic processes, and cell communication. Enriched cellular components were mainly associated with the membrane. Most enriched molecular functions were associated with ion binding, molecular transducer activity and protein binding. As we expected, overrepresentation analysis as well as the network expansion analysis showed that most of the significantly expressed genes were associated with sensory responses.

## Discussion

We analyzed RNAseq data generated from the antennae of *G. m. morsitans* to understand gene regulation in response to either repellent (δ-nonalactone) or attractant (Ɛ-nonalactone) as compared to the untreated controls. Downstream analysis of the entire gene set (13,019 genes) revealed that there was differential expression of various genes in response to the two treatments. We focused on a set of 308 genes that showed significant upregulation due to exposure to an attractant or repellent. The identified chemosensation genes such as gustatory receptors, ionotrophic receptors, odorant binding proteins and Odorant receptors give the fly a situational awareness of the surrounding environment. For example, Gr21a is a potential CO_2_ receptor (Jones et al., 2007) while the highly conserved Ir25a is a co-receptor for Ir21a and together they mediate thermotransduction (Ni et al., 2016). Putrescine and spermidine which are foul smelling amines generated by bacterial degradation of arginine are detected by Ir41a (Silbering et al., 2011). Their differential expression could therefore be attributed to either the repellent (δ-nonalactone) or the attractant (Ɛ-nonalactone) used as a treatment in the experiment.

WGCNA is statistical model that is data and works even on non-model organisms (Degli Esposti et al., 2019). Therefore, when we used WGCNA on *G. m. morsitans* data, we were able to reduce the dimensionality of the data and to extract meaningful patterns using network centrality measures. The resulting co-expression network was useful in selecting genes with significant connectivity patterns that are biologically meaningful. Node degrees helped us identify two genes (CAH3 and Ahcy) that had a degree of 11 and therefore these can be regarded as the top hub genes. In *Drosophila melanogaster*, CAH3 is a carbonate dehydratase involved in generation of protons and bicarbonate from carbonic acid (Overend et al., 2016). *Ahcy* is involved in methionine biosynthesis and metabolism (Brosnan & Brosnan, 2006). The observed betweenness centrality measures mean that some of the genes such as *Teh*2 and *Ir21a* which had values of 1 and 0.8 respectively would have more control over the network as compared to those with lower values. Therefore genes/nodes with high betweenness centrality values are more biologically informative in a module (Riquelme Medina & Lubovac-Pilav, 2016). Closeness centrality measures for majority of the nodes were between 0.1 and 1, which means the resulting network was closely connected, while twenty-seven of the nodes in the network had a clustering coefficient of 1 an indication of complete node connection (Liu, 2018).

Association rule mining enabled us to identify itemset patterns based on the RNAseq genes expression patterns. The first 22 rules indicate that there is a relationship among the genes (itemsets) expression in each condition (transaction) with the following genes *CG18480, CG31663, Ir84a, CG17572, CG5273, Gr63a, Or88a, Or49b, Or2a, Or56a, Or42a, CG3679, Adgf-A, NtR, teq, NimB4, Scp1*, CG3168, CG6126, *Ppn, vkg, LanB2, Tsf1, scf, Sp7* and *Idgf4* always being up or downregulated in response to either repellent (δ-nonalactone) or attractant (Ɛ-nonalactone). The identified rules in this study are biologically significant based on the concept that similar items as in market basket analysis appear together is clearly shown in our results. For example, where the genes CG18480, CG31663, CG17572, CG5273 and CG3679 referred to as consequents (right side) were up (highly expressed), all the genes on the rule antecedent (left side) were also up. The rest of the rules can be interpreted in a similar manner. Genes *Ir84a, Gr63a, Or88a, Or49b, Or2a, Or56a* and *Or42a* are involved in chemosensation in insects. However, genes CG18480, CG31663, CG17572, CG5273 and CG3679 that are co-expressed with the chemosensation genes have no assigned biological function. We therefore hypothesize that these genes also play a role in chemosensation due to their co-expression and association with chemosensory genes. Only two genes (CG3168 and CG6126) were associated with the top non-chemosensory genes that were upregulated due to exposure to an attractant. We used a lift value greater than 2 in generating the rules because values greater than 1 indicate that consequent and antecedent are dependent on one another (Hahsler, Grün & Hornik, 2007).

## Conclusions

We analyzed RNA-Seq data from the antennae of *Glossina morsitans morsitans* (Tsetsefly) after exposure to either a repellant (δ-nonalactone) or an attractant (ε-nonalactone). After pre-processing the data using several methods and software tools to filter out low quality reads and low counts, we proceeded to carry out co-expression network analysis which facilitated the feature reduction by filtering out genes based on the node degree. We found a set of genes occurring in the two treatments and then attempted to find out if these genes could assist us predict the function on uncharacterized genes based on association rule mining. Results from rule mining showed that some of the genes were related based on their appearance in the same itemsets as consequent and antecedent. Most interesting is that we were able to predict the function of some uncharacterized genes in the network. Genes CG18480, CG31663, CG17572, CG5273, CG3679, CG3168 and CG6126 are designated as uncharacterized proteins in the NCBI database. From the association rule analysis, we presume that genes CG18480, CG31663, CG17572, CG5273 and CG3679 have a potential function in chemosensation due to their association with characterized chemosensory genes. Therefore, application of WGCNA and association rule mining is a powerful approach for dimensionality reduction and selecting informative features based on their relationship in the network and eventually the rules. This can assist in predicting the role of biological features with no known function.

## Additional Information and Declarations

### Data Availability

The following information was supplied regarding data availability: The sequences are available at Sequence Read Archive (SRA): PRJNA343267.
